# Ruptured innominate artery pseudoaneurysm presenting as hoarseness in Behçet’s syndrome: a case report

**DOI:** 10.1186/s13256-022-03662-7

**Published:** 2022-11-24

**Authors:** Hamed Ghoddusi Johari, Seyed Arman Moein, Saeedeh Shenavande, Armin Amirian, Sara Sadat Nabavizadeh

**Affiliations:** 1 Trauma Research Center, Thoracic and Vascular Surgery Research Center, Shiraz University of Medical Sciences, Shiraz, Iran; 2 Student Research Committee, Shiraz University of Medical Sciences, Shiraz, Iran; 3 Thoracic and Vascular Surgery Research Center, Shiraz University of Medical Sciences, Shiraz, Iran; 4 Division of Rheumatology, Department of Internal Medicine, Shiraz University of Medical Sciences, Shiraz, Iran

**Keywords:** Innominate artery, Behçet’s syndrome, Pseudoaneurysm

## Abstract

**Background:**

Vascular involvement is an infrequent clinical manifestation of Behçet’s syndrome. Owing to the rarity of arterial involvement in Behçet’s syndrome, there is limited experience in managing this phenomenon.

**Case presentation:**

Here, we report a 28-year-old Iranian man with a Behçet’s syndrome background, who presented with shoulder pain and hoarseness. Chest computed tomography angiography was conducted with a suspicion of a vascular pathology causing pressure on the recurrent laryngeal nerves. The patient was diagnosed with a ruptured innominate artery pseudoaneurysm. An innominate artery to the right common carotid artery bypass was performed, and the pseudoaneurysm was excised and replaced with an expandable polytetrafluoroethylene graft. Eventually, the patient was discharged after an uneventful hospital course.

**Conclusion:**

It appears that we are still a long way from finding the optimal treatment for Behçet’s syndrome vascular involvement, and a combination of surgical and medicinal treatments is required.

## Background

Behçet’s syndrome (BS), as a variable vessel vasculitis, is more common in the Silk Road countries than in other parts of the world. Clinical presentations vary diversely depending on which organs are involved. In contrast to cutaneous and mucosal involvement, vasculitis is relatively rare. The primary form of vasculitis is venous involvement, particularly deep vein thrombosis [1]. On the other hand, in arterial vasculitis of BS, pulmonary artery aneurysms, thrombosis, and extrapulmonary arterial aneurysms/pseudoaneurysms have been reported [2]. Overall, innominate artery aneurysm is a rare condition comprising about 3% of all supra-aortic aneurysms [3].

In this article, we describe an extremely rare presentation of BS and further share our experience in the surgical management of this case.

## Case presentation

The patient was a 28-year-old Iranian man with a history of BS, who presented with right shoulder pain and hoarseness 1 week prior to admission. Going through his medical records, we realized that he had undergone an open right subclavian artery bypass for acute right upper extremity ischemia caused by thrombosis in the aneurysmal right subclavian artery to the distal arterial branches around 1.5 years ago. However, follow-up imaging suggested occlusion in the bypass area. Given the history of recurrent oral aphthous lesions and extensive body folliculitis lesions, BS was suspected. The BS diagnosis was confirmed by a positive human leukocyte antigen (HLA)-B*51 test, an erythrocyte sedimentation rate (ESR) of 79, and a C-reactive protein (CRP) of 5.2 (normal: 0.5), as well as pathological findings from the open right subclavian artery bypass surgery. Cyclophosphamide therapy was the treatment of choice for the patient, but due to infertility concerns, he refused. Therefore, he was treated with azathioprine 100 mg daily, prednisolone 30 mg daily, colchicine 1 mg daily, and rivaroxaban 20 mg daily postoperatively.

The patient’s compliance was good, and according to the latest rheumatological reports prior to his admission to our facility, his BS was under control and no hypercoagulable symptoms were observed.

**Fig. 1:**
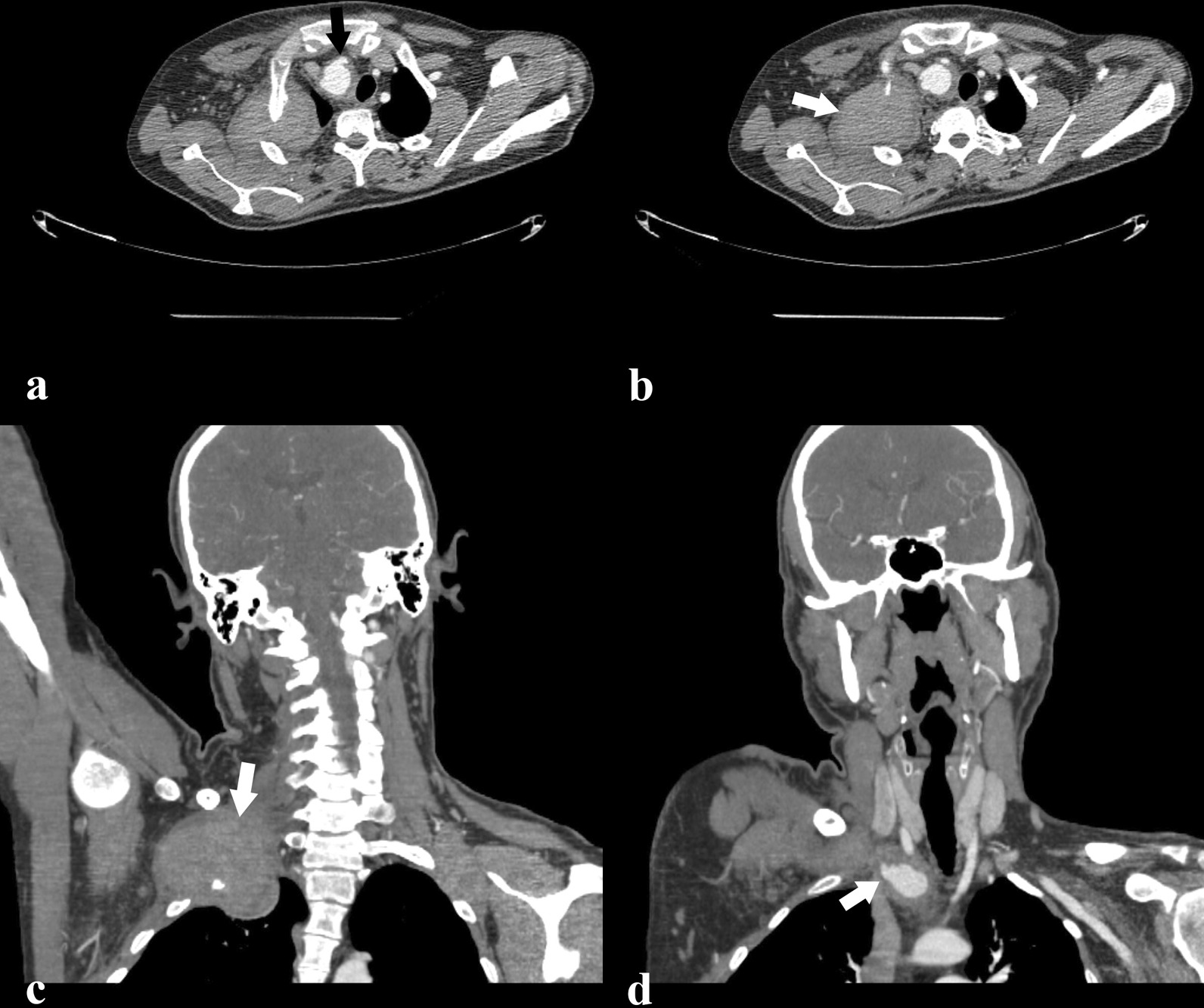
Preoperative contrast-enhanced computed tomography angiogram (CTA). **a** Axial image: the pseudoaneurysm of the innominate artery causing pressure effect on the trachea (black arrow). **b** Axial image: the ruptured pseudoaneurysm hematoma (white arrow). **c** Coronal reconstruction: a large hematoma is observed in the right shoulder extending to the extrapleural area in the apex of the right thoracic cavity (white arrow). **d** Coronal reconstruction: innominate artery pseudoaneurysm is observed (white arrow). It must be noted that the right subclavian artery is occulted

**Fig. 2:**
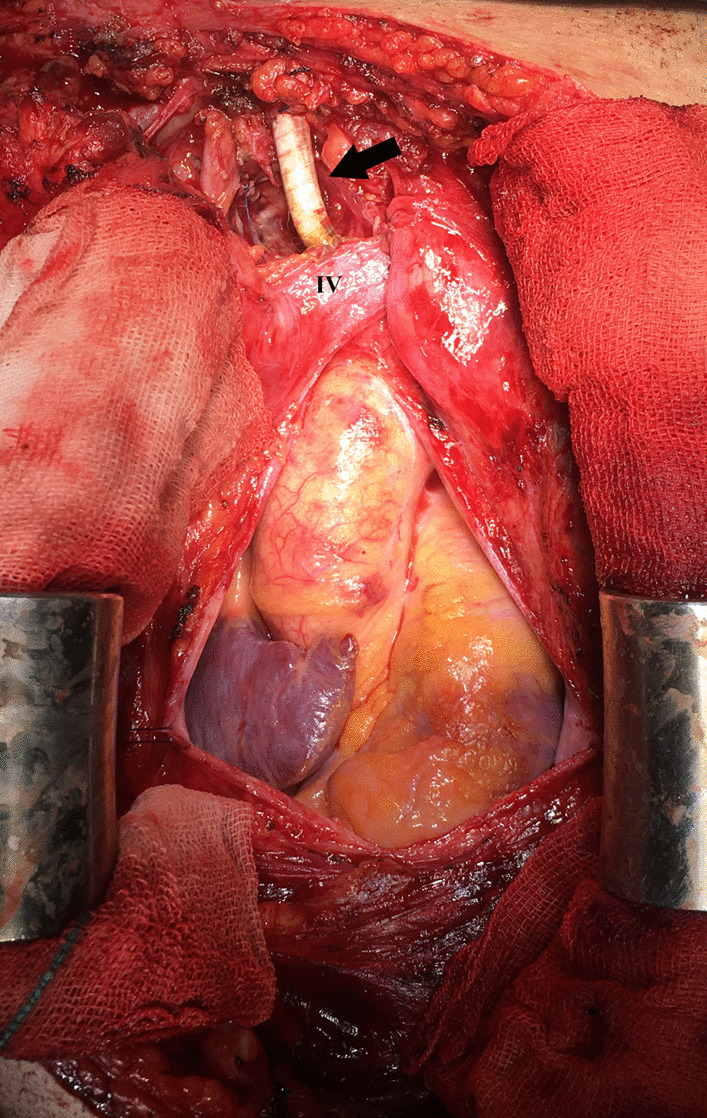
Intraoperative view. Expandable polytetrafluoroethylene (ePTFE) graft bypasses the innominate artery to the right common carotid artery (black arrow). The graft passes right behind the left innominate vein (IV)

After admitting the patient, with all his past history in mind, we requested a contrast-enhanced chest computed tomography angiography (CTA) with suspicion of a vascular pathology causing pressure effect on the recurrent laryngeal nerves. CTA revealed a 32 × 27 mm ruptured pseudoaneurysm originating from innominate artery bifurcation with a 90 × 61 × 65 mm hematoma extending to the right shoulder and forming an extrapleural hematoma at the apex of the right thoracic cavity (Fig. 1). The trachea was deviated to the left side due to the mass effect of the pseudoaneurysm. Despite total occlusion of the right subclavian and axillary arteries by thrombosis, the patient had no complaint regarding his right upper extremity, which shows that there was probably sufficient collateral blood perfusion. Even though we retrospectively reexamined his previous CTA from the former admission, no anomaly was found in the innominate artery. We decided to repair the ruptured pseudoaneurysm by an endovascular approach. Preoperatively, the patient received methylprednisolone to decrease postoperative complications. In a hybrid operation room, we performed a digital subtraction angiography. Unfortunately, owing to the lack of proper landing zone, we could not perform an endovascular repair by stent graft. Inevitably, we approached the pseudoaneurysm by a median sternotomy with supraclavicular extension. After excising the ruptured pseudoaneurysm and evacuating the surrounding hematoma, we performed an innominate artery to the right common carotid artery bypass with a 6 mm expandable polytetrafluoroethylene (ePTFE) graft, which passed right behind the innominate vein (Fig. 2). The pressure effect of the pseudoaneurysm was removed from the right recurrent laryngeal, phrenic, and vagus nerves with no neural damage. We decided not to perform a right subclavian artery bypass since it was previously thrombosed, and the patient had no complaint concerning the right upper extremity. Postoperatively, the patient developed a deep vein thrombosis at the central venous catheter site in the left internal jugular vein. Later, the patient was transferred to the rheumatology ward. The hoarseness of the patient resolved through the hospital course. Monthly infusion of cyclophosphamide 750 mg was started, and the azathioprine was discontinued. Follow-up during the next year showed no sign of recurrence.

## Discussion and conclusions

BS is a rare condition without a known etiology, which includes multisystem inflammatory presentations. In BS, vascular pathology is assumed to be induced by immunological and inflammatory mechanisms that generate a hypercoagulable state and endothelial damage. The vasculitis causes thrombosis by increasing platelet aggregation and inhibiting fibrinolysis [4]. In addition, vasculitis causes deterioration of the media layer, arterial dilatation, and fibrosis, which destroys the vasa vasorum and results in transmural necrosis and subsequent pseudo aneurysm formation or artery wall rupture [5].

Vascular involvement of BS, particularly arterial vasculitis, consists of aneurysm or pseudoaneurysm formation and thrombosis. Contrary to venous vasculitis, arterial involvement of BS usually requires surgical intervention besides high dose prednisolone and cyclophosphamide or anti-tumor necrosis factor(TNF)-α agents [6, 7]. Depending on the location of the arterial involvement, various interventions may be performed. However, the main problem with arterial vasculitis in BS is the recurrence of the condition after proper treatment. In a retrospective study of 25 cases of definite BS with arterial involvement by Tuzun *et al.*, despite medical and surgical therapy, two cases developed aneurysms at new arterial sites after an average of 2 years, and three patients experienced aneurysmal recurrence in the surgical site 6 months postoperation [8]. Although our patient was medically managed to prevent new vascular pathology, he had developed a new pseudoaneurysm in the innominate artery.

There is still no consensus on whether the endovascular or open surgical approach is best in the arterial vasculitis of BS. Endovascular surgery offers a less invasive procedure with probably lower risks of procedure-related mortality. However, in a study by Kwon *et al.* including 12 BS patients with abdominal aortic aneurysm, it was concluded that treating the patients with graft interposition has a lower postoperative aneurysmal recurrence rate than the endovascular approach (14.3% versus 40%) [9].

Various surgical approaches have been practiced to manage innominate artery aneurysms, such as endovascular surgery and partial or full median sternotomy with or without anterior neck and supraclavicular fossa dissection [10].

There is limited experience in managing innominate artery aneurysms in BS patients. In a review of the literature, we only found two similar cases. The first description of this rare presentation of BS was in 1987 by Hamza *et al.* [11]. Furthermore, in 2016, Tsuda *et al.* presented a BS patient with an innominate artery aneurysm that underwent axillo-axillary bypass by PTFE graft interposition. Later, an innominate artery stent graft was deployed through the right common carotid artery [12].

In our case, the subclavian artery was occluded, and the patient had no complaints regarding his right upper extremity. Hence, we avoided any attempt to reconstruct the right upper extremity circulation. Considering the rarity of this condition and the limited experience in managing this presentation, it is not clear whether endovascular or open surgery would be superior. However, the endovascular approach is preferred due to minimal invasiveness. In our case, proper stent graft was unavailable owing to the anatomy of the pseudoaneurysm. Short-interval follow-ups are strongly recommended looking for any signs of recurrence of the aneurysm. New signs and symptoms must be carefully examined for possible further vascular involvement. Hoarseness should be considered as a possible sign of vascular pathology in the mediastinum. Furthermore, the role of proper immunosuppressive medical therapy must not be underestimated. Recent studies show promising outcomes in patients who received immunosuppressive medical therapy before and after the surgical intervention [13].

In addition, there is still controversy regarding the use of anticoagulants in the treatment of BS. There is a concern that anticoagulants may increase the risk of aneurysmal bleeding and hematoma formation in BS patients [14]. In our case, it seems that using anticoagulants led to large hematoma formation after the rupture of the pseudoaneurysm. In BS, thrombosis usually occurs secondary to local endothelial activation due to vasculitis rather than a primitive coagulation disorder. These medications may help prevent thromboembolism formation at the aneurysmal site and prevent end arterial microembolization and gangrene [15, 16]. Therefore, while warfarin and direct oral anticoagulants are used to manage hypercoagulability in patients with BS, direct oral anticoagulants also appear to reduce the risk of major bleeding in routine practice[17, 18]. On the other hand, Vautier *et al.* suggested the use of direct oral anticoagulants in case of deep vein thrombosis (DVT) in BS due to the lower risk of bleeding, as it may also synergize with the rest of the medication, preventing further vascular pathologies [19]. Therefore, by considering all aspects, we chose to add rivaroxaban to the immunosuppressive medication.

Finally, we believe that effective therapy for vascular involvement in BS is still a long way off, and a combination of medicinal and surgical techniques is required. Moreover, additional research is required before the appropriate management of arterial vasculitis in BS can be determined.

The patient consented to the use of related medical history and imaging for educational and scientific purposes.

## Data Availability

All data generated or analyzed during this experimental study are included in this published article.

## Abbreviations

BS: Behçet’s syndrome
HLA: Human leukocyte antigens
ESR: Erythrocyte sedimentation rate
CRP: C-reactive protein
CTA: Computed tomography angiography
DOA: Direct oral agents
DVT: Deep vein thrombosis
ePTFE: Expandable polytetrafluoroethylene
